# m6A Methylation Modification: Perspectives on the Early Reproduction of Females

**DOI:** 10.3390/biom15081102

**Published:** 2025-07-31

**Authors:** Yan Yang, Zhanhong Zheng

**Affiliations:** College of Animal Science and Technology, Jiangxi Agricultural University, Nanchang 330045, China; yangyan23@stu.jxau.edu.cn

**Keywords:** m6A, uterine receptivity, embryo implantation, decidualization

## Abstract

This review examines the regulatory role of m6A methylation modification in embryo implantation, focusing specifically on its impact on uterine receptivity and decidualization. It offers a comprehensive examination of the essential theoretical research on m6A methylation, clarifying its molecular mechanisms and roles in uterine receptivity and decidualization. Furthermore, this review examined the effects of m6A methylation on endometrial-related diseases and early embryonic development. It synthesizes early findings and recent advancements in m6A methylation studies. Through a comprehensive analysis of relevant studies, this review offers novel insights into the molecular mechanisms underlying embryo implantation and suggests potential strategies for assisted reproductive technologies and the treatment of related disorders, thereby serving as a comprehensive reference for future research in this domain.

## 1. Introduction

Infertility has recently become a prominent societal concern, with its global prevalence increasing due to a multitude of factors, including advancing age, occupational influences, substance abuse, environmental pollution, sexually transmitted infections, and lifestyle choices [1]. This condition is increasingly affecting individuals at younger ages [2]. The increasing incidence of infertility can be attributed to multiple factors, with embryo implantation failure being a primary contributor. This study offers a comprehensive review of the regulatory role of m6A methylation modification in embryo implantation, with a specific focus on its impact on uterine receptivity and decidualization. The m6A modification is facilitated by a multi-component methyltransferase complex, comprising METTL3 and METTL14, among others, and its dynamic alterations are crucial for embryonic development [3,4]. Demethylation is mediated by fat mass and obesity-associated protein (FTO) and ALKBH5. Recent studies have demonstrated that METTL3 and METTL14 are essential in establishing uterine receptivity and decidualization [5,6]. These findings offer novel insights into the molecular mechanisms of embryo implantation and propose potential strategies for enhancing assisted reproductive technologies and treating related disorders. Despite the advancement achieved, the specific regulatory mechanisms of m6A modification in uterine receptivity and decidualization require further exploration. Furthermore, the formulation of pharmacological interventions or therapeutic strategies targeting m6A modification-related pathways holds promise for improving embryo implantation success rates and advancing reproductive health.

Reproduction is a complex process encompassing various stages, including spermatogenesis, oogenesis, fertilization, embryo implantation, decidualization, placental development, and pregnancy maintenance [7,8]. Embryo implantation is a critical and complex process essential for the successful establishment of pregnancy in mammals, requiring high-quality embryos and a receptive endometrium [9,10,11]. Despite the relatively high success rates of in vitro fertilization in assisted reproductive technologies, the embryo implantation rate remains suboptimal [12]. Furthermore, insufficient implantation is often the underlying cause of numerous pregnancy complications [13]. Recent research has clarified the crucial regulatory role of m6A methylation modification in embryo implantation, highlighting its importance in this process. This review comprehensively examines relevant studies, exploring the function of m6A methylation modification in embryo implantation, with a specific emphasis on recent advancements in our understanding of uterine receptivity and decidualization.

## 2. The Basic Theory of m6A Methylation Modification

N6-methyladenosine (m6A) represents the most prevalent form of mRNA modification in eukaryotic cells, with each mRNA molecule estimated to contain 3–5 m6A sites, which exhibit dynamic and reversible regulatory characteristics [14]. Empirical evidence demonstrates that m6A modification serves as a potent regulator of gene expression [15]. The m6A modification process is enabled by a multi-component methyltransferase complex, comprising methyltransferase-like 3 (METTL3) and 14 (METTL14), along with additional components, including Wilms’ tumor 1-associated protein (WTAP), RNA-binding motif protein 15 (RBM15s), virus-like m6A methyltransferase-associated protein (VIRMA, also known as KIAA1429), zinc finger CCCH-type containing 13 (ZC3H13), and METTL16 [16]. During m6A methylation, WTAP initially regulates METTL3 and METTL14, forming a complex, and, concurrently, WTAP can catalyze the transfer of methyl groups [17]. Subsequently, RBM15S binds to the complex, directing it to specific RNA sites for m6A modification. Methylation commonly occurs in the 3′ untranslated region (3′-UTR) and proximal to the stop codon [18]. The m6A sites facilitated by METTL16 are predominantly located within introns or at intron–exon junctions, which contrasts with the typical m6A sites found in untranslated regions [19].

**Figure 1:**
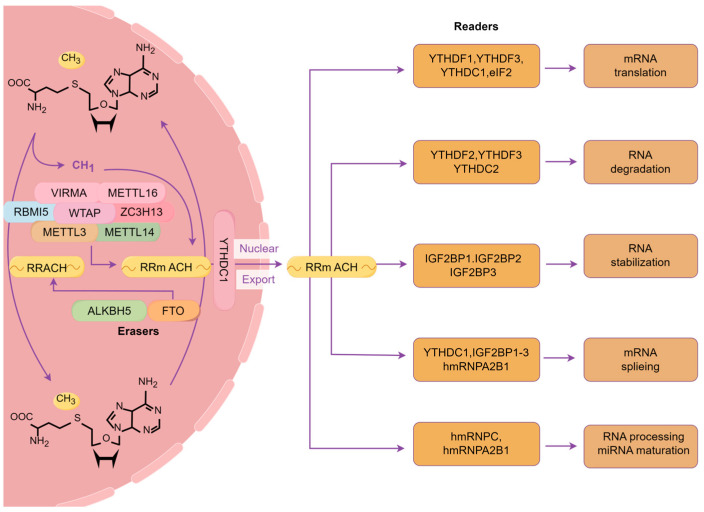
The mechanism of m6A RNA methylation. The dynamic and reversible formation of m6A synthesis is facilitated by distinct m6A regulatory proteins: the “writers” catalyze the addition of m6A marks, the “erasers” selectively remove them, and the “readers” specifically recognize and bind to m6A marks.

The demethylation process is facilitated by two distinct demethylases: FTO and α-ketoglutarate-dependent dioxygenase alkB homolog 5 (ALKBH5) [20]. FTO was identified as the first demethylase in 2011 [21]. The catalytic domain of ALKBH5 can remove m6A methylation from single-stranded RNA (ssRNA) and single-stranded DNA (ssDNA) [22]. Moreover, upon modification, mRNA is identified and acted upon by reader proteins, primarily including YTH domain-containing proteins (YTHDC1/2 and YTHDF1/2/3), insulin-like growth factor 2 mRNA-binding proteins (IGF2BP1/2/3), and eukaryotic translation initiation factor 3 (EIF3) [23]. The five YTH domain-containing proteins can directly recognize the modified mRNA and modulate the degradation and translation efficiency of these RNAs [24,25]. Conversely, IGF2BPs enhance the stability and storage of their target mRNAs in an m6A-dependent manner, consequently influencing gene expression [26]. Furthermore, EIF3 directly binds a single 5′-UTR m6A and recruits the 43S complex to commence translation in a cap-dependent manner (Figure 1) [27]. Recent studies have demonstrated that m6A is essential in various biological processes [28,29].

The modification of m6A methylation has emerged as a viable therapeutic target for numerous diseases. In the progression of non-alcoholic fatty liver disease to hepatocellular carcinoma, METTL3 serves as a therapeutic target, particularly when combined with immune checkpoint blockade (ICB) therapy [30]. Another study has demonstrated that mice with a specific METTL14 knockout in beta cells exhibit reduced m6A levels, replicating the phenotype of human type 2 diabetes. These mice exhibit early onset of diabetes and mortality resulting from decreased beta cell proliferation and depletion of insulin granules. Targeting beta cell-specific m6A methylation transferases with existing therapeutic drugs may provide a novel strategy to enhance beta cell survival and function [31]. Furthermore, regarding reader proteins, studies have demonstrated that YTHDF1 impairs anti-tumor immunity through the m6A-p65-CXCL1/CXCR2 axis, thereby facilitating the initiation and progression of colon cancer. Therefore, YTHDF1 is a potential therapeutic target for ICB therapy [32]. Another study has demonstrated that YTHDF2 expression is upregulated in intrahepatic cholangiocarcinoma (ICC) tissues. Silencing YTHDF2 results in reduced cell proliferation and enhanced apoptosis, highlighting its oncogenic and cisplatin-desensitizing properties. This finding offers novel insights for developing combination therapy strategies for ICC [33]. Regarding eraser proteins, FTO inhibits TNIP1 mRNA expression by removing the m6A modification from TNFAIP3 interacting protein 1 (TNIP1) mRNA. This action activates NF-κB and other inflammatory factors, exacerbating retinal vascular leakage and acellular capillary development. The sustained expression of Tnip1 after intravitreal injection of adeno-associated virus ameliorates endothelial injury, suggesting that the FTO-TNIP1-NF-κB network offers a potential therapeutic target for diabetic vascular complications [34]. Furthermore, under angiotensin II (Ang II)-induced hypertension, cardiac macrophages derived from circulating monocytes predominantly undergo macrophage-to-myofibroblast transition (MMT), associated with increased expression of the RNA m6A demethylase ALKBH5. The macrophage-specific knockout of ALKBH5 reduces Ang II-induced MMT, subsequently enhancing cardiac fibrosis and dysfunction. RNA immunoprecipitation sequencing identifies interleukin-11 (IL-11) mRNA as a target of ALKBH5-mediated m6A demethylation, resulting in enhanced IL-11 mRNA stability and protein levels, offering a reference for potential therapeutic targets for cardiac fibrosis [35]. These findings highlight the crucial role of m6A modification in numerous diseases and provide potential therapeutic targets and strategies.

## 3. The Theoretical Basis of Uterine Receptivity and Decidualization

Embryo implantation requires blastocysts with implantation ability and a uterus in a receptive condition. Endometrial receptivity refers to the state in which the endometrium permits embryo adhesion and invasion during the implantation window, initiating a series of changes in the endometrium and promoting embryo implantation. This receptive state of the endometrium is precisely regulated by two ovarian hormones: 17β-estradiol (E2) and progesterone (P4). These hormones exert their effect through the estrogen receptor (ESR) and the progesterone receptor (PGR), respectively [36]. The female uterus undergoes cyclical changes, transitioning through the proliferative phase, secretory phase, and shedding phase during each menstrual cycle among women of reproductive age. The proliferative phase, also referred to as the follicular phase, is characterized by the growth of multiple ovarian follicles. Under the influence of E2 secreted by these follicles, the endometrium undergoes proliferative changes. In the early proliferative phase, the uterine glands are small and few. During the proliferative phase, epithelial and stromal cells undergo continuous division and proliferation, leading to the thickening of the endometrium to a thickness of 3–5 mm by the end of the phase. At this stage, ovulation occurs as the mature follicle in the ovary releases an ovum, transitioning the endometrium from the proliferative to the secretory phase. During the secretory phase, the corpus luteum develops, and under the influence of P4, the endometrium continues to proliferate. Glandular activity increases, with glands becoming enlarged and tortuous. Subsequently, endometrial stromal cells begin to differentiate, and the uterus gradually enters the receptive phase. In the absence of a pregnancy signal, the uterus transitions to the shedding phase. During this phase, the menstrual corpus luteum in the ovary degenerates, leading to decreased levels of estradiol E2 and progesterone P4. If a blastocyst capable of normal implantation is present in the uterus, and embryo implantation occurs, the pre-decidual cells formed during the late secretory phase will further develop and enlarge under the influence of P4 secreted by the pregnancy corpus luteum, transforming into decidual cells and sustaining the pregnancy. The duration for the uterus’s receptivity is limited, necessitating the embryo to develop into a blastocyst capable of implantation within this period. Concurrently, abnormalities in the epithelial and stromal cells of the endometrium may lead to unsuccessful embryo implantation [37]. In mouse models, on day 1 (D1, the day the vaginal plug is observed) and day 2 (D2) of pregnancy, the uterus, regulated by E2, commences the proliferative phase. On days 3 (D3) and 4 (D4), P4 regulates proliferation termination and the initiation of differentiation in uterine epithelial cells, signifying the onset of the endometrial receptive period [37].

A blastocyst poised for implantation initially interacts with endometrial epithelial cells during the uterus’s receptive state. Influenced by P4, these epithelial cells cease proliferation and commence differentiation, undergoing several transformations, including the reduction of cell microvilli and the loss of cell polarity, to enable normal embryo implantation [38,39]. Ki-67 serves as a marker molecule to signify the receptive condition of the uterus [40].

During implantation, stromal cells proliferate and differentiate, transitioning from fibroblast-like cells to polygonal cells with enhanced secretory functions, in a process termed decidualization. Simultaneously, the extracellular matrix undergoes remodeling, which is accompanied by angiogenesis. The maintenance of decidual tissue function is crucial for establishing a microenvironment conducive to embryo implantation and subsequent stable development. During this process, stromal and decidual cells secrete several growth and immunomodulatory factors, which are essential for the transformation of endometrial epithelial cells, the activation of immune cells, and other associated processes. Abnormal decidualization of stromal cells is a significant contributing factor to recurrent miscarriage [41]. The decidualization of endometrial stromal cells is essential in embryo implantation and the maintenance of embryonic development.

The interaction between endometrial epithelial cells and stromal cells is essential for embryo implantation and subsequent post-implantation development. Previous studies indicate that E2 promotes endometrial epithelial cell proliferation by binding to estrogen receptor alpha (ERα) in endometrial stromal cells and through paracrine signaling mechanisms. Concurrently, heart and neural crest derivatives expressed protein 2 (HAND2), a basic helix–loop–helix transcription factor, inhibiting fibroblast growth factor expression within endometrial stromal cells. This inhibition affects the P4-PR signaling pathway, consequently inhibiting the proliferation of epithelial cells without affecting stromal cell proliferation [42]. HAND2 acts as an inhibitor of the epithelial cell E2 signaling, thereby preparing the uterine epithelium for embryo implantation. The lack of HAND2 in the uterus led to compromised embryo implantation, indicating that HAND2 within the stroma modulates embryo implantation through a P4-induced epithelial differentiation process [40].

Additionally, the Indian hedgehog (IHH) signaling molecule, a downstream target of the PGR, is significantly expressed in the uterine luminal epithelium of wild-type mice before embryo implantation [43]. IHH binds to the PTCH1 receptor in uterine stromal cells, resulting in COUP-TFII expression (NR2F2). This induction subsequently enhances the expression of bone morphogenetic protein 2 (BMP2) and Wnt family member 4 (WNT4), facilitating stromal cell proliferation [44]. Concurrently, COUP-TFII may contribute to preserving the equilibrium between ESR and PGR signaling pathways [45]. These findings demonstrate the complex and meticulously regulated interactions between the endometrial epithelium and stroma, influenced by hormonal influences.

## 4. The Regulation of m6A Methylation on Preimplantation Embryo Development

**Figure 2:**
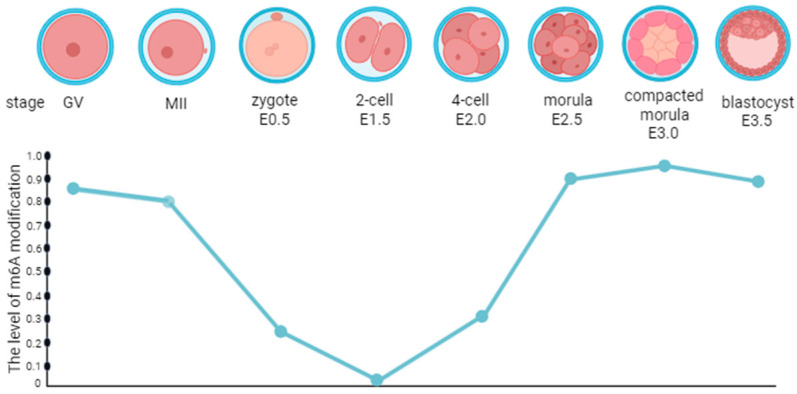
M6A undergoes dynamic changes during the development of preimplantation embryos in mice.

Embryo implantation necessitates an embryo with implantation ability and a uterus in the receptive phase. Furthermore, 6A is essential in many physiological and pathological processes. Significant alterations in genetic material occur during early embryonic development, including m6A methylation modifications [46]. Notably, m6A modifications exhibit dynamic changes before mouse embryo implantation, with elevated levels being observed at the blastocyst stage compared to the two-cell, four-cell, and eight-cell stages [47,48]. In *Drosophila melanogaster*, the m6A level increased significantly during the early stage of embryonic development but decreased sharply 2 h after fertilization, remaining low in the subsequent embryonic development and early larval stage [49]. In pigs, m6A methylation persists from the zygote stage to the blastocyst stage, exhibiting a marked increase during the transition from morula to blastocyst [50]. During embryonic development, the dynamic changes in m6A may correlate with gene expression reprogramming in this process. Meanwhile, differences in genomes, developmental processes, and epigenetic regulatory mechanisms among different species may explain the variations in m6A levels during early embryonic development across species. During preimplantation embryo development, m6A undergoes dynamic changes (Figure 2) [51].

The alterations in m6A modification and its regulatory factors are essential in embryogenesis and critical for embryonic development. A previous study demonstrated that METTL3 knockdown in mature germinal vesicle (GV) oocytes of female mice significantly impeded oocyte maturation, primarily due to reduced mRNA translation efficiency. This disruption affected the maternal-to-zygotic transition and zygotic genome activation, presumably by obstructing the mRNA degradation mechanism [52]. Furthermore, the knockout of Mettl14 in mice from embryonic day 6.5 caused marked embryonic growth retardation, primarily attributed to resistance to differentiation, leading to embryonic lethality in early pregnancy [4]. Previous studies have demonstrated that in mice, YTHDC1 expression is low in GV oocytes but significantly elevated in MII oocytes and 1-cell, 2-cell, and 4-cell embryos [53]. Conversely, another study demonstrated that a single Ythdfs in zebrafish is not essential for maternal mRNA clearance or development timing [54]. Embryos lacking FTO demonstrated delayed development, with maternal loss of FTO significantly impairing decidual formation and the production of embryos at the E7.5 stage [55]. Moreover, ALKBH5 knockdown in human trophoblasts enhanced trophoblast invasion, whereas ALKBH5 overexpression inhibited this cellular invasion [56].

## 5. m6A Modification in the Early Stages of Female Reproduction

The human endometrium permits embryo implantation during the implantation window. Endometrial receptivity disorders can lead to several reproductive conditions, including infertility caused by complete implantation failure; abortion caused by inadequate implantation; and pregnancy complications, including preeclampsia caused by poor implantation [57]. Despite advancements in preimplantation genetic diagnosis technologies that have markedly enhanced embryo quality, instances of implantation failure continue among women of reproductive age undergoing in vitro fertilization–embryo transfer. This evidence further highlights that reduced endometrial receptivity is a primary factor contributing to recurrent implantation failure (RIF). During endometrial decidualization, the proliferation and apoptosis of decidual cells occur simultaneously, maintaining a dynamic balance and supporting the growth and development of embryos after implantation. This balance is closely related to placental formation and the sustenance of normal pregnancy. Decidualization defects or abnormalities can lead to RIF, recurrent spontaneous abortion (RSA), endometriosis, and other related diseases [58]. Some studies have demonstrated that the pregnancy outcome of patients with RIF is primarily affected by endometrial status, which is closely related to endometrial dysfunction and changes in expression profiles [59,60,61]. Simultaneously, previous research has identified that the endometrium in patients with RSA exhibits “hyperreceptivity,” with impaired decidualization in endometrial stromal cells. This disruption leads to the prolongation and premature opening of the endometrial implantation window, compromising the embryo selection process and leading to the implantation of non-high-quality embryos. Therefore, complications, including spontaneous abortion, can occur, adversely affecting pregnancy outcomes [62].

RNA methylation as a form of epigenetic regulation is an emerging research field, and its significance in regulating cell growth and differentiation is increasingly recognized. Uterine progesterone resistance, resulting from reduced expression of the progesterone receptor (PGR) protein, is implicated in the pathogenesis of endometriosis [63], adenomyosis [64], and endometrial cancer [65]. Numerous studies have demonstrated that several endometrial-related diseases are associated with m6A modification (Table 1). In cases of endometriosis, METTL3 expression and m6A levels in ectopic and eutopic endometrial tissues were significantly lower than those in the normal control group [66,67]. METTL3 significantly inhibits the endometriosis progression by increasing cell senescence through the SIRT1/FOXO3 signaling pathway [68]. METTL3 and m6A expression levels in the endometrium of individuals with adenomyosis are significantly lower than those in healthy controls [69]. A marked decrease in m6A level is observed in endometrial cancer, which is attributed to METTL14 mutation or the decreased METTL3 expression [70,71].

**Figure 3:**
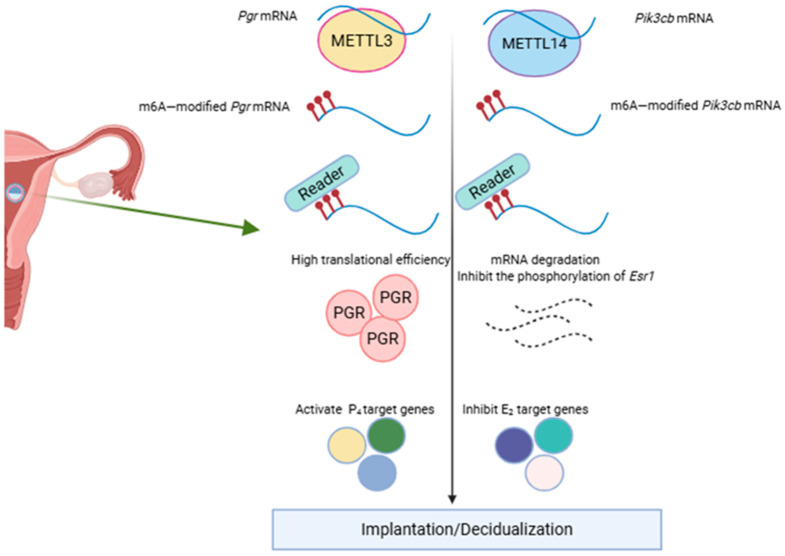
The role of Mettl3 and Mettl14 in embryo implantation.

These results indicate that m6A methylation modification is essential in the regulation of endometrial function. Subsequent studies by Zheng et al. have demonstrated that conditional METTL3 knockout in mouse endometrium can result in embryo implantation failure, manifested as early pregnancy embryo loss and impaired uterine receptivity. Furthermore, the loss of METTL3 significantly reduced PGR expression, which adversely affected normal progesterone signaling. Furthermore, the loss of METTL3 significantly reduced PGR expression, disrupting normal progesterone signaling. In the artificial decidualization model, METTL3 deficiency results in a complete loss of decidual response, manifested by a decrease in decidualization marker gene expression, including PRL and IGFBP1. Additionally, METTL3 is essential for maintaining the stability and translation efficiency of Pgr mRNA through m6A-mediated translation control, a process dependent on YTHDF1. This finding highlights the critical role of METTL3 in implantation. Notably, the METTL3-PGR axis is conserved between mice and humans, indicating its potential involvement in related disease pathogenesis. This highlights the significance of the METTL3-PGR axis in uterine pathophysiology [5]. Kobayashi et al. reported that the conditional METTL14 deletion in the mouse endometrium similarly results in implantation failure, characterized by the loss of embryos during early pregnancy and impaired uterine receptivity. In addition, the METTL14 deletion markedly enhanced tErα phosphorylation, while the PGR signaling pathway was unaffected. In the artificial decidualization model, the lack of METTL14 leads to a complete loss of decidual response, manifested by a decrease in decidualization marker gene expression, including PRL and IGFBP1. They demonstrated that METTL14 is essential for maintaining normal embryo implantation and decidualization by regulating the ERα signaling pathway and innate immune response [6]. These findings highlight the distinct regulatory roles of METTL3 and METTL14 in the endometrium, consistent with those of observations previously reported in mouse embryos [3,4,74], embryonic stem cells [75], the small intestine [76,77,78], and the testis [79]. Despite their divergent functions, the deletion of Mettl3 or Mettl14 leads to similar phenotypic outcomes (Figure 3). These results highlight the complex and subtle role of m6A modification in the regulation of uterine function.

## 6. Conclusions and Future Perspectives

The natural conception rate for women each menstrual cycle is approximately 30%, primarily attributed to implantation failure [80]. Numerous studies have demonstrated the significant role of m6A modification in embryo implantation. This study provides new insights into the molecular mechanisms of embryo implantation and provides potential new strategies for treating assisted reproductive technology and associated diseases. Through in-depth analysis of the m6A modification regulatory network, combined with the advancement of clinical application methods and the integration of new technologies, it is possible to enhance reproductive health and solve related diseases.

However, there is a paucity of research regarding the development of endometrial receptivity and decidualization in the context of m6A. Previous studies have clarified the regulatory roles and mechanisms of METTL3 and METTL14 in facilitating endometrial receptivity and decidualization. The dynamic alterations in m6A modification during early embryonic development highlight its significant impact on embryo quality and implantation capability. METTL3 and METTL14, as primarily m6A methyltransferases, are essential in maintaining the signal transduction of the PGR and ERα, influencing endometrial receptivity and decidualization. However, other factors associated with m6A modification warrant further study to enhance our understanding of the molecular networks governing embryo implantation and decidualization. Simultaneously, m6A modification and its associated regulatory factors are potential therapeutic targets for various diseases. However, current research largely remains within the realms of basic studies. The primary animal model for studying human embryo implantation is the mouse, which shares approximately 85% genomic homology with humans, indicating good genetic similarity. Furthermore, some human embryo implantation-related diseases can be modeled in mouse models. For instance, uterine-specific knockout of Bmi1 can mimic the pathological features of RIF in humans [74]. Moreover, the mouse functions as a highly mature animal model for studying embryo implantation, with a well-established technical framework. However, it has several limitations. Many mechanisms related to embryo implantation are not conserved between mice and humans; hence, a therapeutic strategy that proves efficient in mice may not necessarily be effective in humans. Nevertheless, numerous therapeutic approaches remain untested in humans.

Clarifying the exact regulation mechanism of m6A modification during embryo implantation is of high significance for future research. Certain studies have demonstrated that there is an interaction between m6A modification and other epigenetic modifications under specific physiological or pathological conditions. For instance, in sepsis-associated acute lung injury, histone lactic acidosis induces ferroptosis through METTL3-mediated m6A modification [81]. In advanced clear cell renal cell carcinoma (ccRCC), METTL14-mediated m6A modification enhances the mRNA stability and zinc finger protein 14 (ZFP14) expression, subsequently enhancing K48-linked ubiquitination, facilitating the degradation of signal transducer and activator of transcription 3 (STAT3), and thereby inhibiting ccRCC progression [82].

Consequently, a comprehensive understanding of the interplay between m6A modification and other epigenetic modifications during embryo implantation and decidualization is crucial. This understanding will clarify the role and significance of m6A modification in these processes, thereby offering a robust theoretical foundation for its application in embryo implantation and associated domains. Additionally, using the existing research results, the formulation of drug intervention or treatment strategies for m6A modification-related enzymes can improve the success rate of embryo implantation and bring new hope to individuals experiencing infertility.

In conclusion, future research should focus on clarifying the specific regulatory mechanism of m6A modification in early pregnancy and developing corresponding clinical applications to enhance reproductive health and solve related diseases.

## Figures and Tables

**Table 1:** M6A modification-related enzymes involved in some endometrial diseases.

Events of Endometrial Diseases	M6A Modification-Related Enzymes	References
RIF	METTL3, YTHDF1	[5,72]
RSA	METTL14	[73]
Endometriosis	METTL3, YTHDF2	[66,67,72]
Endometrial cancer	METTL3, METTL14	[70,71]
Uterine adenomyosis	METTL3	[69]

## Data Availability

No new data were created or analyzed in this study. Data sharing is not applicable to this article.
